# Neurovascular microcirculatory vasodilation mediated by C-fibers and Transient receptor potential vanilloid-type-1 channels (TRPV 1) is impaired in type 1 diabetes

**DOI:** 10.1038/srep44322

**Published:** 2017-03-13

**Authors:** P. Marche, S. Dubois, P. Abraham, E. Parot-Schinkel, L. Gascoin, A. Humeau-Heurtier, PH. Ducluzeau, G. Mahe

**Affiliations:** 1 Endocrinology Department, University Hospital of Angers, Angers, France; 2 Laboratory of Vascular Investigations, University Hospital of Angers, UMR CNRS6214/INSERM1083, LUNAM Université, Angers, France; 3 Methodology and Biostatistics Unit, University Hospital of Angers, Angers, France; 4 University of Angers, LARIS - Laboratoire Angevin de Recherche en Ingénierie des Systèmes, Angers, France; 5 Endocrinology Department, University Hospital of Tours, Tours, France; 6 INSERM Clinical Investigation Center (CIC 14 14), Rennes, France, Université de Rennes 1 and LUNAM University, Inserm 1083/CNRS 6214, Faculty of Medicine, Angers, France

## Abstract

Microvascular dysfunction may have an early onset in type 1 diabetes (T1D) and can precede major complications. Our objectives were to assess the endothelial-dependent (acetylcholine, ACh; and post-occlusive hyperemia, PORH), non-endothelial-dependent (sodium nitroprusside, SNP) and neurovascular-dependent (local heating, LH and current induced vasodilation, CIV) microcirculatory vasodilation in T1D patients compared with matched control subjects using a laser speckle contrast imager. Seventeen T1D patients - matched with 17 subjects according to age, gender, Body-Mass-Index, and smoking status - underwent macro- and microvascular investigations. The LH early peak assessed the transient receptor potential vanilloid type 1 channels (TRPV1) mediated vasodilation, whereas the plateau assessed the Nitirc-Oxyde (NO) and endothelium-derived hyperpolarizing factor (EDHF) pathways. PORH explored sensory nerves and (EDHF), while CIV assessed sensory nerves (C-fibers) and prostaglandin-mediated vasodilation. Using neurological investigations, we observed that C-fiber and A-delta fiber functions in T1D patients were similar to control subjects. PORH, CIV, LH peak and plateau vasodilations were significantly decreased in T1D patients compared to controls, whereas there was no difference between the two groups for ACh and SNP vasodilations. Neurovascular microcirculatory vasodilations (C-fibers and TRPV 1-mediated vasodilations) are impaired in TD1 patients whereas no abnormalities were found using clinical neurological investigations. Clinicaltrials: No. NCT02538120.

Over 200 million people have diabetes worldwide, 10% of whom suffer from Type 1 diabetes (T1D). This disease preferentially affects young adults, and can lead to several complications such as sensorimotor neuropathy and macroangiopathy^1^. Diabetic neuropathy is one of the most common chronic complications of diabetes, affecting more than 50% at the advanced stage of the disease (more than 20 years of evolution)^2^. This sensorimotor polyneuropathy can lead to diabetic foot ulcer, and in some cases, to Charcot disease (neuroarthropathy of the foot). Unfortunately, in the natural history of the disease, the neuropathy remains asymptomatic over a long period, inducing delayed diagnosis^3^. The rate of lower limb amputation is however 15 times higher in diabetic patients than in nondiabetic patients, and more than 50% of diabetic amputees require subsequent amputation of the contralateral limb within 4 years of loss of the first leg^4^. Moreover, macroangiopathy related to atherosclerosis is a major cause of morbidity and mortality in diabetic patients^5^. Endothelial dysfunction, while discussed in terms of vascular beds (conductance artery *vs* resistance artery) and the stage of the disease^6^,^7^, may appear early on in the disease process and contributes to the induction and development of atherosclerotic plaques. Indeed, the endothelium plays a major role in blood flow regulation by producing several substances, such as nitric oxide (NO), prostacyclin, and endothelium-derived hyperpolarizing factor (EDHF), and participates in the control of many vascular functions: modulation of vasomotor tone, coagulation, cell adhesion mechanisms and migration, inflammatory processes, remodeling mechanisms, and vascular permeability^8^. Detecting all these complications as early as possible to improve patient management thus represents a major challenge for clinicians.

It has long been thought that microcirculation abnormalities in diabetes precede major complications^9^. Cutaneous microcirculation is considered as a mirror of generalized vascular function and is a useful translational model for investigating mechanisms of cutaneous physiology and cutaneous pathophysiology due either to skin disease or to other diseases such as diabetes^10^. Various microvascular tests for some of these, though not validated, have been used to assess both neurovascular abnormalities and endothelial dysfunction in T1D patients^4^,^6^,^7^,^11^,^12^,^13^,^14^,^15^,^16^,^17^,^18^. The heterogeneity of both tests used and groups of patients studied does not allow us to understand the changes to the microvascular mechanisms involved in T1D or the time course over which microvascular abnormalities develop. And yet several dedicated tests are available and have been well-described for assessing the various mechanisms of vasodilation such as the NO-mediated pathway, the EDHF-mediated pathway and neurovascular pathways, whether mediated or not by transient receptor potential vanilloid Type 1 channels (TRPV-1)^19^. This latter pathway is of particular interest since it has been suggested that TRPV-1 may play a major role in the occurrence of T1D^20^,^21^ and in cardiovascular mortality^22^. We hypothesized that microvascular abnormalities, especially impaired TRPV-1 mediated vasodilation, can be found in type 1 diabetes.

The aims of the present study were therefore to use five dedicated microvascular tests to assess neurovascular functions and endothelial functions in T1D patients compared to matched control subjects, and to determine the time course of the microvascular abnormalities.

## Results

Seventeen young adults with T1D (age 27.4 (20.0–36.8), 41% male, duration of diabetes 9.3 ± 7.8 years) and 17 controls matched for age, BMI, smoking status, and sex (age 24.6 (22.9–24.6), 47% male) were studied. All the diabetic patients were treated with insulin (insulin pens or subcutaneous insulin pump). We did not note any significant difference between the diabetic and control groups for blood pressure, pulse wave, and lipid levels (see Tables 1 and 2). Furthermore, as expected, fasting glycemia (8.3 (6.6−11.7) mmol/l *vs*. 4.7 (4.5−4.9) mmol/l, *p* = 0.0006) and glycosylated hemoglobin (8.4 (7.7−9.5)% *vs* 5.3 (5.2−5.5)%, *p* < 0.0001) were higher in the diabetic group than in the control group. Moreover, skin temperature at baseline and during each experiment was not significantly different between control and T1D subjects.

### Neurological investigations

Our results show no difference on the upper limb and the lower limb for any of the parameters studied between T1D and controls, except for the diapason at the hallux and the vibration perception at the foot and hand: for them, the thresholds were significantly higher for T1D (see Table 3).

### Microvascular investigations

We found similar baseline vascular conductance in control subjects and T1D patients, whatever the microvascular investigations. Moreover, no statistical difference was observed between the two groups for the peak value observed after ACh and for the peak or plateau observed after SNP iontophoresis (see Table 4).

**Figure 1:**
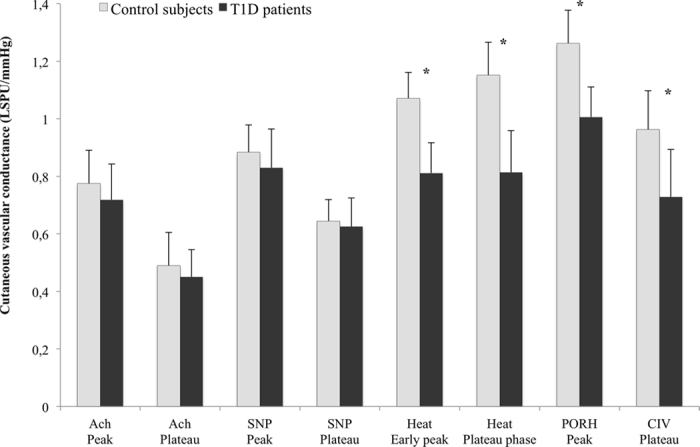
Mean cutaneous vascular conductance (CVC: LSPU/mmHg) in type 1 diabetic patients in response to each microvascular stimulation compared to control subjects. Bars represent the mean ± SD. LSPU means Laser Speckle Perfusion Unit. *p < 0.05.

Furthermore, the peak obtained during PORH was significantly lower in the T1D group than in the control subjects (1.01 ± 0.21 LSPU/mmHg and 1.26 ± 0.23 LSPU/mmHg, respectively, *p* = 0.003). Similarly, the peak (within 7 minutes following the start of heating) and the late plateau after heating (22 minutes following the start of recording) were both significantly decreased in the diabetic group compared to controls (0.81 ± 0.21 LSPU/mmHg and 1.07 ± 0.18 LSPU/mmHg for the peak; 0.81 ± 0.29 LSPU/mmHg and 1.13 ± 0.23 LSPU/mmHg for the plateau, respectively, *p* = 0.0004 and *p* = 0.002) (Fig. 1).

For the CIV test, we did not note any difference in vascular conductance at rest between the T1D patients and the control subjects (see Table 4). However, the mean plateau value observed after the second 5 s cathodal current application was significantly lower in the T1D patients than in the matched controls (0.73 ± 0.33 LSPU/mmHg and 0.96 ± 0.27 LSPU/mmHg, respectively, *p* = 0.03).

## Discussion

Our study, which compared five specific microvascular responses at the forearm level between T1D patients and control subjects matched for age, sex, BMI and smoking status, shows that neurovascular responses are significantly impaired in T1D patients with normal microalbuminuria and without macrovascular abnormalities compared with control subjects, whereas ACh-response and non-endothelial-dependent (ie, SNP) response are preserved.

Diabetic polyneuropathy is linked to impaired large-diameter, myelinated (A-alpha and A-beta) fibers and small-diameter, unmyelinated nerve (A-delta and C) fibers^16^. Our clinical neurological results show that impairment, which was only found with A-beta fiber tests, is higher at the lower extremity level than at the upper extremity, confirming that nerve injury begins at the lower extremities, moving up to the upper extremities^17^. It has been suggested by previous authors that small-diameter, unmyelinated damage, especially C-fiber damage, appears earlier than large-diameter, myelinated (A-alpha and A-beta) fibers^16^. In our study using neurological investigations, C-fiber and A-delta fiber functions in T1D patients were similar to control subjects, whereas microvascular neurovascular responses were impaired in T1D patients compared with control subjects. This point is in accordance with Krishnan *et al*. whose work showed that C-fiber dysfunction assessed by “LDI flare” can be detected earlier than with clinical neurological investigations in type-2 diabetic (T2D) patients^23^.

Our study shows that the axon reflex response assessed by two different neurovascular tests (peak local heating and cathodal CIV) is significantly impaired in T1D. As described elegantly by Minson *et al*. and Wong *et al*., during the local thermal hyperemia test, the axon reflex mediates the early peak through transient receptor potential vanilloid type 1 (TRPV-1) channels located on sensory nerves, whereas the late plateau is dependent on NO. (50–60%) and EDHFs (40–50%)^19^,^24^. In our study, the significantly diminished peak response to local thermal hyperemia could suggest an abnormality (less activity) of the TRPV-1 channels at the skin level in T1D patients compared to control subjects. An important role of TRPV-1, which is distributed in the peripheral nervous system and in the network of sensory neurons that innervate the pancreatic islet, has already been evoked in T1D^20^,^25^. In the nerves of the islet beta cells, TRPV-1 can modulate T cell function. Inhibition of TRPV-1 has been shown to protect mice from autoimmune diabetes^21^, whereas TRPV-1 hypofunction initiates local inflammation and an autoimmune process in the pancreas leading to insulin resistance and beta cell destruction in non-obese diabetic mice^25^. Razavi *et al*. have explained this controversy by a local feedback interaction between sensory nerves and beta cells^25^. In 2013, Sadeh *et al*. also demonstrated that TRPV-1 might be a susceptible gene for T1D patients in a Jewish population^26^, although this result needs to be confirmed in other populations. Interestingly, using TRPV-1 immunochemistry, Wilder-Smith *et al*. found increased keratinocyte TRPV-1 expression in diabetics compared to controls^27^. We hypothesize that this overexpression could be linked to hypofunction of TRPV-1 that would induce a diminished microvascular peak response in our T1D patients, but this remains to be studied. In our study we also used the cathodal CIV, a second axon reflex test that is partially mediated by prostaglandins^28^. This test is based on the anodal CIV test that has been widely used and studied in the literature^29^,^30^. Using this test, we confirm that T1D patients have an impairment of this response, suggesting either an impairment of the axon reflex response (C-fibers) or prostaglandin release as in T2D patients^29^. As the late plateau phase of the ACh test is also partially mediated by prostaglandins^30^ and was not different from the control subjects, we assume that the impairment of the CIV response in T1D patients is due to an impairment of the axon reflex response (C-fibers). This corroborates the results obtained with the local thermal peak suggesting a lesser activity of TRPV-1 channels. This lesser activity of TRPV-1 could also be a risk factor for cardiovascular diseases^22^.

Our study also demonstrates that the PORH peak is significantly reduced in T1D patients compared to control subjects. Several other authors have found the same results in T1D and T2D^31^, whereas others have found no difference between diabetics and control subjects^15^,^32^ and, in one study, a surprising increase in PORH response^33^. The lack of difference between diabetics and control subjects could be due to a higher age of the population studied^32^ or a higher duration of diabetes^15^ that may have minimized the difference between the populations. The test used in our study enables us to assess the sensory nerves, the large-conductance calcium activated potassium (BK_CA_) channels and EDHF, especially the 5,6-epoxyeicosatrienoic acid pathway^34^,^35^. This microvascular response is mediated neither by NO nor by prostaglandins^35^. The decrease of the PORH peak in T1D patients could suggest a sensory nerve dysfunction, but also an EDHF dysfunction since the local thermal hyperemia plateau, that was decreased in our T1D patients is also partially mediated by EDHF^24^. Several authors have shown that the EDHF pathway is altered in diabetes^36^,^37^,^38^. This decrease of the local thermal plateau response can be widely found in the literature but the mechanism involved in such a decrease remains to be clarified^12^,^14^,^32^,^39^.

In our study, the ACh response, which is considered to be a specific test for endothelial function^10^, is similar between T1D patients and control subjects and is in accordance with previous publications^14^,^17^,^40^,^41^. Contrary to our results, Khan *et al*.^12^ showed a significantly reduced response to ACh in T1D. Two explanations can be given: i) skin blood flow measurements were not standardized for blood pressure whereas there was a difference between the systolic pressure of the diabetics compared to the controls; and ii) the subjects’ skin temperature was lower (30 °C) than in our study (33.8 °C) and this parameter is known to modify the microcirculatory response^42^,^43^. Gomes *et al*.^15^ also found a decreased response to ACh, although the difference was not significant for an electric charge of 2 mC, which is the one utilized in our study. Most of the other studies that have found a difference in ACh responses between diabetics and controls, used a higher electric charge (between 6 mC to 16 mC) or studied older populations^6^,^12^,^13^.

Furthermore, we did not find a significant difference in response to SNP that explores the endothelial-independent pathway between T1D patients and controls. This NO donor acts directly on smooth muscle cells to induce relaxation through an increase in cGMP formation^44^. These findings with this electric charge (2 mC) are consistent with previous studies^6^,^12^,^15^,^18^,^45^ and demonstrate the integrity of the smooth muscle cell function. Other authors found a decrease in SNP response, though using a higher electric charge or in older populations^13^,^14^.

Lastly, several studies were previously performed to evaluate microvascular abnormalities in T1D patients. Most of these studies assessed one, two and up to four microvascular vasodilatory pathways at the same time, limiting the knowledge of the time course for the onset of microvascular abnormalities^7^,^11^,^12^,^14^,^46^. According to our results, neurovascular response impairment seems to appear earlier than ACh or SNP responses and before impairment of macrovascular abnormalities. Therefore, for a clinician, the choice would have to focus either on the local thermal test, the cathodal CIV test or the PORH test in order to assess microvascular dysfunction.

Our study has some limitations: The menstrual cycle stage was not checked in this study. This might have influenced the results since the role of the female hormonal status in skin reactivity is discussed^47^. The proportion of females was not, however, statistically different between the groups. Moreover, while the absence of microangiopathy in T1D was confirmed by negative microalbuminuria, we had no information on diabetic retinopathy.

In conclusion, this study suggests that: i) neurovascular responses at the forearm level are impaired in T1D patients without impairment of the ACh and SNP responses compared with matched control-subjects; ii) microvascular response mediated by TRPV-1 channels is diminished; and iii) neurovascular abnormalities appears earlier than other microvascular abnormalities.

## Methods

### Subjects

Seventeen T1D patients were recruited in the diabetic care unit of the Angers University Hospital and matched with 17 healthy volunteers according to age, gender, Body Mass Index (BMI) and smoking status. Before their participation, all subjects were thoroughly informed of the methods and procedures and gave their written consent to participate in this prospective study approved by the “Comité Protection des Personnes-OUEST II” ethics committee (Number: 2012-A010008-35 and acceptance date: 2012). Informed consent was obtained from all subjects. Experiments were carried out in accordance with the Declaration of Helsinki. The protocol was registered in the American National Institute of Health database under reference No. NCT02538120 (August, 2015).

Each subject had to meet the following inclusion criteria: be at least 18 years old, have read and signed the informed consent document. Diabetic subjects had to display at least one diagnostic criterion according to World Health Organization (Fasting plasma glucose ≥126 mg/l (7 mmol/l) twice after 8 h of fasting, or casual plasma glucose ≥200 mg/l (11.1 mmol/l) in presence of hyperglycemia symptoms (polyuria, polydipsia and unexplained weight loss), or 2-hour glucose ≥200 mg/l after an oral glucose tolerance test (OGTT) with 75 g glucose), and at least one positive antibody from among: ICA (against islet cytoplasm), GAD (against glutamic acid decarboxylase), IA2 (against tyrosine phosphatase) and IAA (anti-insulin). Healthy volunteers had glycated hemoglobin (HbA1c) equal to or lower than 5.6%. All treatments were allowed, except for anti-inflammatory drugs during the study and in the 10 days preceding inclusion, as they are known to abolish current-induced vasodilation (please refer to the microvascular tests section)^29^.

### Protocol

Each diabetic subject or volunteer was required to complete 2 distinct visits. The first one constituted the inclusion visit during which a medical examination (height, weight, BMI, blood pressure, tendon reflexes, Ankle Brachial Index [ABI]) and an interrogation searching for cardiovascular risk factors were performed. If the subject accepted to be included in the study, he/she was asked to sign the consent document and blood tests were conducted at the end of the first visit. The second visit consisted in neurological and microvascular investigations.

### Clinical neurological investigations

The presence or absence of neuropathy was tested in accordance with the recommendations of the San Antonio Conference (1988) using the Neuropathy Symptom Score (NSS) and the Neuropathy Disability Score (NDS) evaluating large myelinated fibers. Both scores range from zero (absence of neuropathy) to ten^48^. A technician ipsilateral to the vascular investigations performed neurological investigations. The quantitative evaluation was carried out by assessing the vibration perception threshold (VPT) with a neurothesiometer (Case-IV, Medical Electronics Co., USA) exploring A beta fibers. The VPT was also tested with a Rydel-Seiffer fork (64 Hz) exploring A beta fibers. The heat and cold detection thresholds (HDT and CDT) as well as the heat and cold pain thresholds (HPT and CPT) were tested (TSA II, Medical Electronics Co., USA), on the dorsum of the foot and hand examining C fibers and A delta fibers^49^. For each measurement, the starting temperature of the probe was 32 degrees Celsius (°C) and the participants were asked to push a button when the test threshold was reached. For analysis, the relative heat deflection temperature, which is obtained by subtracting the skin temperature, was used.

### Macrovascular investigations

Pulse wave velocity was assessed using a Mobil-O-Graph (I.E.M. GmbH, Stolberg, Germany) and the ankle-brachial index (ABI) using a hand-held Doppler on both limbs. The ABI was calculated as the ratio of the highest systolic pressure in the dorsalis pedis or posterior tibial artery to the highest systolic pressure between both arms according to guidelines^50^.

### Microvascular investigations

Experiments were performed with the participants resting in the supine position. All tests were performed in a quiet temperature-controlled room after a 20-minute acclimatization period.

Recently, laser speckle contrast imaging (LSCI) has been developed. LSCI uses the random speckle pattern generated by the illumination of the tissues under study by a coherent laser light. LSCI recordings of the cutaneous blood flow (CBF) in the forearm were performed using a 70-mW system (PeriCam PSI System^®^, Perimed, Järfälla, Sweden) with a laser wavelength of 785 nm^42^. The sampling frequency was 18 Hz. The distance between the laser head and skin surface was fixed at 15 cm taking into account environmental conditions^42^. The software expresses recorded values in laser speckle perfusion units (LSPU).

### Microvascular tests

Five vasodilator microvascular tests involving different physiological vasodilation pathways were simultaneously performed on the same volar aspect of the forearm.

#### ACh iontophoresis (endothelial-dependent pathway)

Iontophoresis is a non-invasive method that drives a pharmacologically-charged drug by electrorepulsion through the interstitium surrounding the blood vessels^10^. Transdermal iontophoresis of ACh (Sigma-Aldrich Corporation, L’Isle d’Abeau, France) was performed with an iontophoresis chamber (LI 611, Perimed Jarfalla, Sweden) and corresponded to the anode. The iontophoresis chamber was filled with ACh (2%) dissolved in deionized water. The peak, which is dependent on the muscarinic receptor, and the late plateau, which is partially prostaglandin-dependent, were studied.

#### SNP iontophoresis (endothelial-independent pathway)

Transdermal iontophoresis of SNP (Nitriate^®^, SERB, Paris, France) was performed with an iontophoresis chamber (LI 611, Perimed Jarfalla, Sweden) and corresponded to the cathode. The iontophoresis chamber was filled with SNP (1%) dissolved in deionized water. The PeriIont Micropharmacology System (Perimed, Jarfalla, Sweden) was plugged into the iontophoresis chamber containing ACh and to the iontophoresis chamber containing SNP and delivered the current. The current stimulation was set to 0.1 mA for 20 seconds.

#### PORH (endothelial-dependent and endothelial-independent pathway)

The PORH for exploring neurovascular interactions and probably the endothelium-derived hyperpolarizing factor (EDHF)^51^ was measured by producing ischemia for 3 minutes with a blood pressure cuff inflated to 50 mmHg over the subject’s systolic blood pressure^51^.

#### Current-induced vasodilation (CIV) (neurovascular pathway and prostaglandin dependent pathway)

Transdermal iontophoresis of deionized water was performed with an iontophoresis chamber (LI 611, Perimed Jarfalla, Sweden) and corresponded to the cathode. The current application was delivered on the active probe in two consecutive 5-second applications separated by 4 minutes^28^. For CIV, we studied the late-plateau response.

#### Local thermal hyperemia (neurovascular and endothelial-dependent pathway)

The hyperemic response to heat was evaluated by using a skin-heating probe filled with deionized water and heated to 44 °C to assess heat-induced vasodilation. Heat-induced vasodilation is due to nerve fibers for the early peak and to the NO-dependent pathway and the EDHF-dependent pathway for the late plateau^24^,^52^.

### Number of subjects

Searching for a difference of at least 0.35 in CIV between the two groups with a SD of 0.30 for each group based on our previous study^29^, the minimum number of subjects to be included for alpha = 0.05 and a 90% power was 16 per group.

### Data and statistical analysis

The descriptive results are presented as the mean +/− standard deviation (SD) for variables with normal distribution and as the median (25 centile–75 centile) when distribution was not normal. Skin blood flow was measured over a region of interest (ROI) and a time of interest (TOI) from images recorded by the LSCI. The size of the ROI was 20 mm^2^. The TOI was set to 5 s for the peak and 10 s for the baseline and plateau measurements^53^. The results for the microvascular recordings (peak or plateau) are expressed as cutaneous vascular conductance (CVC). The CVC was calculated as the ratio of CBF to the mean arterial pressure (mmHg) and expressed in LSPU/mmHg^10^. It has previously been reported that, when using LSCI, expressing the results in CVC is the best way to obtain the lowest coefficient of variation (18.7% with an Intra-Class Correlation coefficient = 0.87 [0.65–0.95]) for the 7-day interval of reproducibility^54^.

The normality of the distribution of variables was tested with the Shapiro-Wilk test. The microvascular results obtained in T1D patients and the control subjects were compared with a T-test when there was normal distribution and with a Mann-Whitney test when the distribution was not normal. Statistical analyses were performed using MedCalc for Windows, version 12.5 (MedCalc Software, Ostend, Belgium). For each statistical analysis, a two-tailed p value ≤ 0.05 was considered significant.

## Additional Information

**How to cite this article**: Marche, P. *et al*. Neurovascular microcirculatory vasodilation mediated by C-fibers and Transient receptor potential vanilloid-type-1 channels (TRPV 1) is impaired in type 1 diabetes. *Sci. Rep.* **7**, 44322; doi: 10.1038/srep44322 (2017).

**Publisher's note:** Springer Nature remains neutral with regard to jurisdictional claims in published maps and institutional affiliations.

## Figures and Tables

**Table 1:** Baseline clinical characteristics of type 1 diabetic patients and control subjects.

Patient characteristics	Control subjects (n = 17)	Type 1 diabetic patients (n = 17)	p
Clinical variables			
Male n (%)	8 (47)	7 (41)	0.73
Smoker n (%)	6 (35)	7 (41)	0.72
Age (years)	24.6 (22.9−24.6)	27.4 (20.0−36.8)	0.39
Duration of diabetes (years)		9.29 ± 7.67	
BMI (kg/m^2^)	21.67 ± 2.64	21.6 ± 2.28	0.94
SBP (mmHg)	113.53 ± 10.38	118.24 ± 10.66	0.20
Heart rate (bpm)	68.59 ± 10.04	70.82 ± 9.73	0.51
Pulse wave velocity (m/s)	4.95 (4.8−5.2)	4.98 (4.7−5.7)	0.69
Right ankle brachial index	1.17	1.16	0.70
Left ankle brachial index	1.15	1.16	0.92

Data presented as mean ± SD, median (interquartile range), or n (%). BMI: body mass index; SBP: systolic blood pressure. Bpm means beat per minute.

**Table 2:** Baseline biological characteristics of type 1 diabetic patients and control subjects.

Patient characteristics	Control subjects (n = 17)	Type 1 diabetic patients (n = 17)	p
Biological variables			
APTT (s)	32 (31−33)	30 (28−31)	0.07
PR (%)	94.12 ± 7.34	93.82 ± 10.16	0.92
Platelets (G/L)	250.4 ± 68.49	276.5 ± 40.97	0.18
MPV (fl)	10.61 ± 0.57	10.28 ± 0.78	0.16
Hb (g/dL)	14.30 ± 1.23	14.02 ± 1.26	0.52
Ht (%)	43.59 ± 2.90	41.61 ± 3.31	0.07
White cells (G/L)	5.42 (5.20−6.76)	5.85 (5.25−7.05)	0.73
Creatinin (μmol/L)	77.18 ± 11.99	67.68 ± 8.54	0.01
CKD-EPI* (mL/mn)	107.5 ± 11.1	114.0 ± 16.1	0.18
LDL (mmol/L)	2.74 ± 0.50	2.91 ± 0.69	0.41
HDL (mmol/L)	1.31 ± 0.32	1.38 ± 0.25	0.77
Triglycerids (mmol/L)	0.79 (0.65−0.87)	0.77 (0.56−0.96)	0.88
Glucose (mmol/L)	4.7 (4.5−4.9)	8.3 (6.6−11.7)	0.0006
HbA1C (%)	5.3 (5.2−5.5)	8.4 (7.7−9.5)	<0.0001
HbA1C (mmol/mol)	34.3 ± 2.5	71.1 ± 21.7	<0.0001
Microalbuminuria (mg/L)	11 (10−17)	10 (6−15)	0.25

Data presented as mean ± SD or median (interquartile range). APTT: activated partial thromboplastin time; PR: Prothrombin ratio; MPV: mean platelet volume; Hb: Hemoglobin; Ht: Hematocrit. *Chronic Kidney Disease Epidemiology Collaboration.

**Table 3:** Neurological characteristics of the populations.

	Control subjects	Type 1 diabetic patients	p
Neuropathy scores			
NSS	0 (0−0)	0 (0−4)	0.30
NDS hand	0 (0−0)	0 (0−0)	1
NDS foot	0 (0−0)	0 (0−1)	0.08
Sensory thresholds			
Vibration hand (AU)	0.92 (0.55−1.24)	1.29 (1.03−2.74)	**0**.**049**
Vibration foot (AU)	1.45 (1.04−1.67)	3.04 (1.79−3.93)	**0**.**003**
Cold detection hand (Δ °C)	1.2 (0.8−1.4)	1.1 (1.0−1.6)	0.94
Cold detection foot (Δ °C)	2.5 (2−3.2)	3.4 (1.8−4.8)	0.23
Cold pain hand (Δ °C)	15.3 (7.6−26.9)	17.3 (9.1−22.5)	0.91
Cold pain foot (Δ °C)	17.4 (8.0−23.8)	18.2 (8.2−22.0)	0.51
Heat detection hand (Δ °C)	2.0 (1.2−2.7)	2.2 (1.4−2.8)	0.78
Heat detection foot (Δ °C)	4.3 (3.2−4.9)	4.0 (2.9−6.2)	0.89
Heat pain hand (Δ °C)	11.3 (8.6−13.5)	11.7 (10.5−13.6)	0.72
Heat pain foot (Δ °C)	11.6 (10.1−14.6)	12.7 (8.3−14.4)	0.74
Diapason hand (AU)	8 (8−8)	8 (7−8)	0.06
Diapason toe (AU)	8 (8−8)	7.5 (7−8)	**0**.**01**

NSS is neuropathy symptom score, NDS is neuropathy disability score. AU means Arbitrary Unit and (Δ °C) means variation from 32 °Celsius.

**Table 4:** Microvascular characteristics of the population.

Microvascular investigations	Control subjects	Type 1 diabetic patients	p
*ACh*			
Base (LSPU/mmHg)	0.34 ± 0.09	0.34 ± 0.09	0.88
Peak (LSPU/mmHg)	0.78 ± 0.23	0.72 ± 0.25	0.49
Plateau (LSPU/mmHg)	0.49 ± 0.23	0.45 ± 0.19	0.73
*SNP*			
Base (LSPU/mmHg)	0.38 ± 0.07	0.34 ± 0.09	0.16
Peak (LSPU/mmHg)	0.88 ± 0.19	0.83 ± 0.27	0.51
Plateau (LSPU/mmHg)	0.64 ± 0.15	0.63 ± 0.20	0.75
*Heat*			
Base (LSPU/mmHg)	0.36 ± 0.11	0.33 ± 0.12	0.48
Early peak (LSPU/mmHg)	1.07 ± 0.18	0.81 ± 0.21	**0**.**0004**
Late plateau (LSPU/mmHg)	1.13 ± 0.23	0.81 ± 0.29	**0**.**0018**
*PORH*			
Base (LSPU/mmHg)	0.44 ± 0.11	0.44 ± 0.15	0.91
Peak (LSPU/mmHg)	1.26 ± 0.23	1.01 ± 0.21	**0**.**0026**
*CIV*			
Base (LSPU/mmHg)	0.34 ± 0.09	0.32 ± 0.08	0.53
Plateau (LSPU/mmHg)	0.96 ± 0.27	0.73 ± 0.33	**0**.**03**
*T° cut (°C*)	33.84 ± 1.44	34.26 ± 1.47	0.4

Data presented as mean ± SD. Ach is Acetylcholine, SNP is Sodium Nitroprusside, PORH is post occlusive reactive hyperemia, CIV is current induced vasodilatation and T° cut is cutaneous temperature. LSPU means Laser Speckle Perfusion Unit.
